# Toward Imaging Defect-Mediated Energy Transfer between Single Nanocrystal Donors and Single Molecule Acceptors

**DOI:** 10.1021/cbmi.3c00015

**Published:** 2023-04-04

**Authors:** Danielle R. Lustig, Zach N. Nilsson, Justin T. Mulvey, Wenjie Zang, Xiaoqing Pan, Joseph P. Patterson, Justin B. Sambur

**Affiliations:** † Department of Chemistry, Colorado State University, Fort Collins, Colorado 80523-1872, United States; ‡ School of Advanced Materials Discovery, Colorado State University, Fort Collins, Colorado 80523-1872, United States; § Center for Complex and Active Materials, University of California, Irvine, Irvine, California 92697-2025, United States; ∥ Department of Chemistry, University of California, Irvine, Irvine, California 92697-2025, United States; ⊥ Department of Materials Science and Engineering, University of California, Irvine, Irvine, California 92697-2025, United States; # Irvine Materials Research Institute, University of California, Irvine, Irvine, California 92697-2025, United States; ∇ Department of Physics and Astronomy, University of California, Irvine, Irvine, California 92697-2025, United States

**Keywords:** defects, single molecule, FRET, super-resolution, ZnO, energy transfer

## Abstract

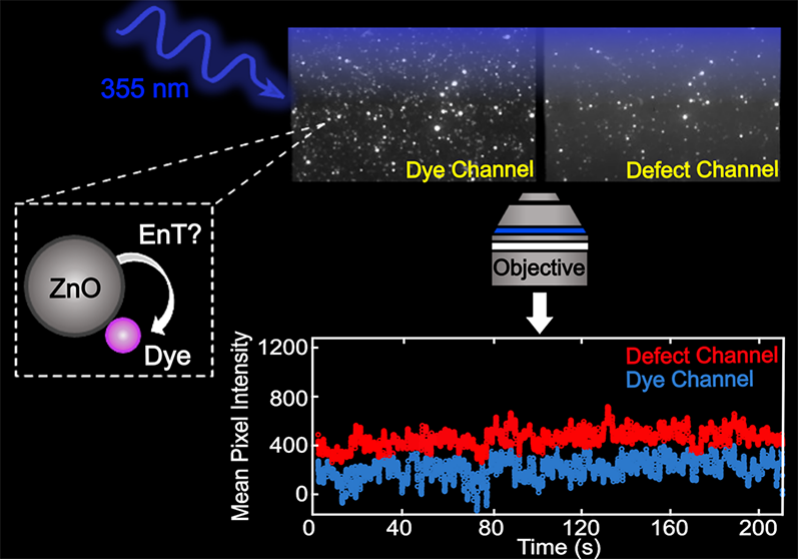

Defect-mediated energy transfer is an energy transfer process between midgap electronic states in a semiconductor nanocrystal (NC) and molecular acceptors, such as fluorescent dye molecules. Super-resolution fluorescence microscopy represents an exciting technique for pinpointing the nanoscale positions of lattice defect sites in, for example, a micrometer-sized particle or thin film sample by spatially resolving the location of the acceptor dye molecules with nanometer resolution. Toward this goal, our group performed ensemble-level, time-resolved fluorescence spectroscopy measurements of ZnO NC/Alexafluor 555 (A555) mixtures and calculated that the emissive defect sites are located, on average, 0.5 nm from the NC surface [NilssonZ. N.; J. Chem. Phys.2021, 154 ( (5), ), 05470433557543 10.1063/5.0034775]. However, ensemble-level measurements cannot spatially resolve the defect sites on single particles, nor can they distinguish between surface-adsorbed dye molecules that participate in the energy transfer (EnT) process from those that do not. In this work, we compared the photoluminescence intensity trajectories of 789 isolated, single ZnO NC donors to those of 73 non-specifically bound and five specifically bound ZnO NC/A555 pairs, where the donor and acceptor centroid positions were separated by a distance that was smaller than our localization precision (40 nm). We observed minor fluorescence intensity fluctuations in the donor and defect channels instead of clear anticorrelated intensity fluctuations, which could be explained by (1) the presence of multiple emissive defect sites per NC, (2) donor–acceptor separation distances slightly larger than the Förster radius (*R*_0_ = 3.1 nm; defined as the distance at which EnT is 50% efficient), and/or (3) poor dipole–dipole coupling. The single molecule imaging methodology we developed, an alternating ultraviolet–visible excitation sequence combined with multicolor photon detection, successfully distinguishes specifically bound and non-specifically bound NC/dye pairs and can be applied to study a wide range of hybrid NC/dye energy transfer systems.

## Introduction

Semiconductor nanocrystal (NC) donors engage in energy transfer (EnT) with molecular acceptors such as organic dyes^1−9^ and metal coordination complexes.^10−16^ In this scenario, an electronically excited semiconductor nanocrystal “donor” transfers its energy to a molecular “acceptor”. When the absorption of the dye acceptor overlaps with the NC bandgap emission, the EnT process has been physically modeled using Förster theory in which singlet exciton transfer occurs via through-space dipole–dipole coupling from the donor dipole located at the NC center to a surface-adsorbed dye acceptor.^17,18^

Aside from band edge electronic states, defect electronic states can participate in the EnT process. Time-resolved spectroscopy experiments have revealed that surface defect sites on PbS NCs can serve as intermediates in triplet–triplet energy transfer.^14,19^ There is also some indication that defect states in CdSe NCs also participate in triplet–triplet energy transfer.^20−22^ Alternatively, Mulvaney and co-workers demonstrated a particularly interesting defect-mediated singlet exciton EnT process between ZnO NCs and AlexaFluor dyes.^23^ The same phenomenon was also observed with Ga_2_O_3_ NCs and ATTO 590 dyes.^24^ Furthermore, defect-mediated energy transfer of ZnO nanowire arrays^25^ and, more recently, thin films^26^ with rare earth ions has been observed. In the singlet exciton defect-mediated process, the energy exchange process occurs between a trapped exciton, located at an energy level inside the forbidden bandgap of the semiconductor, and an acceptor molecule positioned at or near the NC surface.^14,20^ The defect-related energy level is commonly termed the midgap state, a surface defect state, or a surface state in the literature.^14,27−29^

Both the band edge and the defect-mediated EnT process have been physically modeled using Förster resonance energy transfer (FRET) theory,^23,24,30−37^ which is well established for molecular donor/acceptor pairs. Our group showed that ensemble-level time-resolved photoluminescence (TRPL) spectroscopy can reveal the average separation distance between emissive defects in ZnO NCs and surface-adsorbed A555 acceptors when the NC radius is larger than the Förster radius (*R*_0_).^38^ In that study, we discuss how FRET-based analyses cannot reveal the locations of emissive defect sites in semiconductor NCs when *R*_0_ is larger than the NC radius even when the geometry of the NC is taken into account using a restricted geometry model^30^ of NC–molecule EnT.^38^

Unfortunately, ensemble-level measurements are unable to measure the spatial distribution of emissive defects for the following reasons. In general, the error in estimating the number of adsorbed dye molecules per NC is large because the semiconductor NC concentration in a bulk solution is not trivial to measure. Moreover, ensemble-level EnT measurements cannot distinguish which surface-adsorbed acceptor molecules participate in the EnT process. Distinguishing active versus inactive acceptor molecules could help in understanding the donor dipole orientation factor and whether all molecules contribute equally to defect PL quenching, which is an assumption in the stochastic binding model of EnT.^31^ FRET-based models also assume that donor dipoles are located at the NC center (i.e., the point dipole approximation fails),^40−44^ which has important consequences for a related underlying assumption that the dipole orientation factor (κ) is . Because many underlying assumptions in FRET-based models likely do not hold for NC/molecule donor/acceptor pairs, the community is unable to correctly analyze and interpret ensemble-level steady-state and TRPL data.

Single molecule, super-resolution fluorescence microscopy experiments performed at the single NC level could overcome the complications and limitations described above. Single molecule EnT experiments have the potential to pinpoint the locations of emissive defect sites in thin films, large nanostructures, or microwires, assuming the defect sites are spatially separated by a distance larger than the spatial resolution in single molecule fluorescence microscopy experiments (typically 20–40 nm).^45^ At the same time, they distinguish EnT-active from spectator molecules by selectively exciting the donor and acceptor species in the NC/dye conjugate, as Banin and co-workers showed for single CdSe/CdS nanorod donors in the presence of multiple ATTO 590 acceptors.^46^ The authors observed step-like changes in both acceptor and donor emission, enabling them to calculate the energy transfer efficiency and donor–acceptor distances. However, the single molecule, single NC approach has not been applied to study defect-mediated energy transfer. Additionally, the superoptical resolution imaging method has not been applied to dye/NC systems to pinpoint energy transfer events with nanometer spatial resolution. We note that scanning tunneling microscopy and superoptical resolution imaging of nanocrystal emission have been used to image energy flow in nanocrystal solids,^47,48^ but they have not been applied to nanocrystal/molecule systems under solution phase or photocatalytic conditions. In this work, we develop a single molecule, single NC-level imaging methodology to study defect-mediated EnT between semiconductor NCs and molecular dyes.

## Experimental Methods

### NC Synthesis

The 4.5 nm diameter ZnO NCs were synthesized using a base hydrolysis of zinc acetate dihydrate in ethanol following the approach of Wood et al.^49^ First, 1.0 g of Zn(OAc)_2_·2H_2_O (Sigma-Aldrich) was added to 100 mL of 200 proof ethanol (Pharmco-Aaper) in a 250 mL round-bottom flask. The solution was stirred and heated to 68 °C to dissolve the zinc acetate. Then, 2 mL of a 20% methanolic solution of tetramethylammonium hydroxide (TMAOH, Sigma-Aldrich) was added to the flask as quickly as possible. The solution was stirred, and the reaction temperature was maintained at 68 °C for 500 min. Then, the reaction was quenched by injecting a 10 mL aliquot of the reaction mixture into 30 mL of hexanes, causing the NCs to precipitate. The mixture was centrifuged to separate the NCs from the unreacted Zn^2+^ and TMAOH. This washing procedure was repeated five times with hexanes. The washed NCs were suspended in spectrophotometric grade ethanol and stored at −4 °C when not in use. All reagents were used as received without further purification.

### Nanocrystal Characterization

The absorbance and photoluminescence spectra of the ZnO NCs were measured using an HP 8452A diode array spectrophotometer and Edinburgh Instruments FS5 instrument, respectively. All spectra were measured at room temperature in spectrophotometric grade ethanol in 1 cm quartz cuvettes. Transmission electron microscopy (TEM) samples were prepared by drop casting the NCs from a hexane solution onto a lacey carbon grid. The average and standard deviation values for the NC diameter were determined via TEM (JEOL JEM-2100F, 200 keV) of at least 50 NCs. The molarities of the ZnO NC samples were calculated from TEM and elemental analysis following the approach of Yu et al.^50^ and described in detail in our previous work.^38^ Scanning transmission electron microscopy (STEM) characterization was performed on a JEOL Grand ARM 300CF microscope equipped with a cold field emission gun (FEG) and double spherical aberration correctors operated at 300 kV. All of the HAADF-STEM images were recorded with a probe current of 23 pA, using a convergence semiangle of 21 mrad and inner and outer collection angles of 79 and 180 mrad, respectively.

### Single Molecule Fluorescence Microscopy

Details regarding sample preparation, the experimental setup, and image analysis procedures can be found in the Supporting Information. Briefly, quartz slides (Delta Technologies, QS-0310) were immersed in Piranha solution for at least 12 h. The quartz slides were rinsed with 10 MΩ water and stored in spectrophotometric grade ethanol. Approximately 20 μL of the ethanolic ZnO NC solution was spin coated on a clean quartz slide rotating at 1000 rpm. ZnO NC/dye samples were prepared by drop-casting 40 μL of a 0.1 nM dye solution on the quartz slide. The dye droplet remained on the slide for 1 min before the liquid was removed with a nitrogen gas stream. A drop of Immersol W 2010 immersion oil (Zeiss) was placed between the NC/dye-coated quartz slide and a #1 glass coverslip (19904). The Immersol oil was chosen because (1) the NCs and A555 molecules are not soluble in this media and, therefore, the fluid prevents the NCs and molecules from detaching from the quartz slide, (2) the ZnO NC emission decays in air, (3) the ZnO NCs and molecules detach from the slide if the experiments are performed in the same ethanol solvent used for ensemble-level testing, and (4) the Immersol fluid is index-matched to our 60× water immersion objective (NA = 1.2, Olympus, UPLANSAPO60×/W).

The NC/dye-coated quartz slide sample was placed on the stage of an Olympus IX73 inverted microscope equipped with a prism-type total internal reflection fluorescence (TIRF) microscopy setup. Two excitation lasers illuminated the sample. A 355 nm laser (Coherent Genesis CX) selectively excited the ZnO NCs, while a 532 nm laser (Coherent Obis) selectively excited the A555 molecules. A programmable Thorlabs shutter allowed excitation with either one laser or both lasers at the same time. Photons emitted from the sample were collected through the objective, passed through a long-pass filter (cutoff wavelength of 440 nm) in the filter cube of the Olympus microscope, and then directed to an image splitter (Hamamatsu Gemini W-view), which uses a dichroic mirror (cutoff wavelength of 550 nm) to split the photon stream into two channels defined by the bandpass filters used for each channel. One bandpass filter was chosen to match the emission signal from the A555 molecules (585/40 bandpass). This channel represents the “dye channel”. The second bandpass filter was chosen to match the defect emission from ZnO only (490/60 bandpass), forming the “defect channel”. The two channels were projected onto an electron multiplying charge collection device (EM-CCD) camera (Andor iXon 897) operated to produce a single image, split vertically. Figure S1 shows a representative example of the split image before subsequent image processing. Fluorescence images were continuously acquired at a 25 ms frame rate while the excitation lasers were alternated in an on/off fashion, as described in more detail below. The intensity trajectories of single ZnO NCs and dye molecules from the image stacks, or movies, were extracted using the detailed image analysis algorithm described in the Supporting Information (see Figures S2–S5).

## Results

### Ensemble-Level Defect-Mediated EnT between ZnO NCs and A555

**Figure 1:**
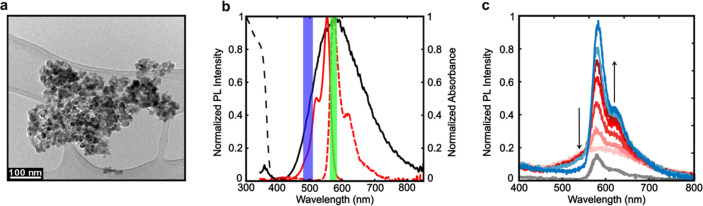
(a) TEM image of ZnO NCs. The average diameter is 4.5 ± 0.67 nm from 50 NCs. (b) Normalized absorption and emission spectra for ZnO NCs (black dashed line and black solid line, respectively) and normalized absorption and emission of the A555 dye (red solid and red dashed lines, respectively). The blue shaded region represents the bandpass filter (490/60 nm) for the “defect channel” in single molecule imaging experiments. The green shaded region refers to the 585/40 nm bandpass filter for the “dye channel”. (c) Steady-state emission spectra of ZnO alone (light pink) and mixtures of ZnO donors with increasing A555 acceptor concentration (0–32 nM) where teal indicates the highest acceptor concentration. The gray spectrum is that of 32 nM A555 dye alone.

We synthesized 4.5 ± 0.67 nm diameter (*N* = 50 NCs) ZnO NCs (Figure 1a) and demonstrated that these NCs participate in defect-mediated energy transfer with A555 molecules using ensemble-level fluorescence spectroscopy. Figure 1b shows the absorption and emission properties of the ZnO NC donors and A555 acceptors. Upon excitation of the ZnO NC bandgap with ultraviolet (UV) light, a broad defect emission peak appears at 600 nm that has been attributed to oxygen vacancies.^23,33,51−54^ This ZnO defect emission peak overlaps completely with the absorption spectrum of A555 acceptors. When 70 nM ZnO NCs were mixed with 32 nM A555 and the bandgap of the ZnO NCs was excited with 355 nm light, the defect PL peak is quenched and the donor fluorescence increases by a factor of 6 beyond the intensity observed in the absence of the donor (Figure 1c, top blue trace vs gray trace). Hence, ensemble-level measurements show clear defect-mediated EnT behavior when the ZnO NC donors and A555 acceptors freely diffuse in ethanol. We recognize that the dye emission overlaps strongly with the broad defect PL, which presents experimental complications such as unambiguously distinguishing photons emitted from A555 acceptors from NC donors. However, the rationale for choosing the A555 acceptor is that this molecule does not absorb the 355 nm excitation laser light used to excite the ZnO NC donors. AlexaFluor dyes with red-shifted emission profiles such as A595 strongly absorb the UV excitation light.

### Single Molecule, Single NC-Level Imaging Methodology

**Figure 2:**
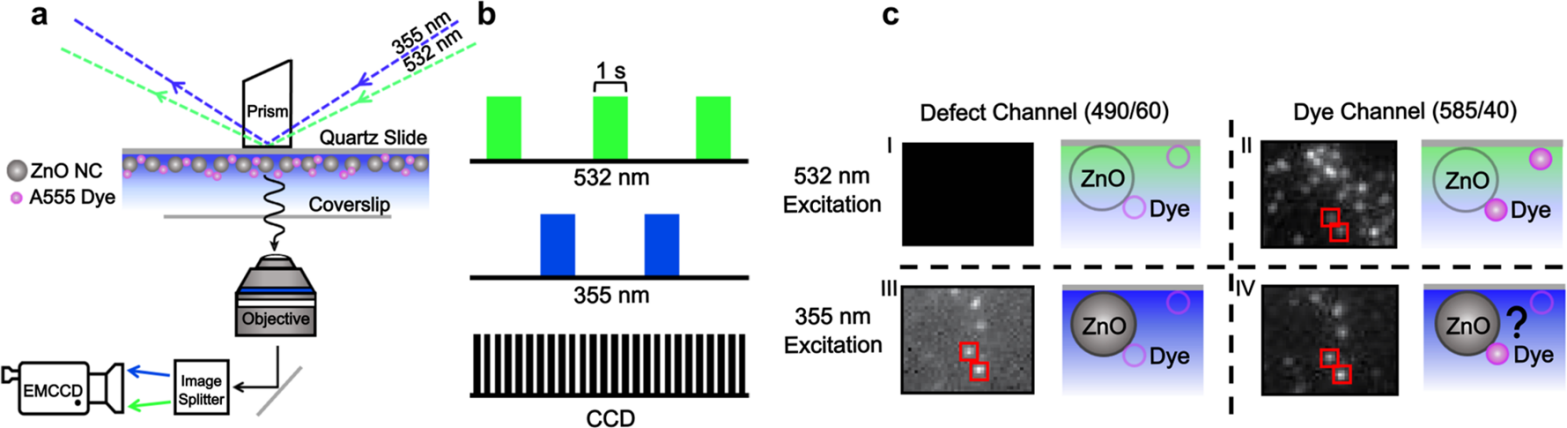
(a) Single molecule fluorescence microscopy of defect-mediated EnT. A 6 mW 355 nm laser excites ZnO NC donors, and a 6 mW 532 nm laser excites A555 acceptors immobilized on a quartz slide. The photons emitted from the sample were sent through an image splitter to produce “dye” and “defect” channels on the EM-CCD camera, defined by the filter sets in Figure 1b. (b) Excitation sequence for the 355 and 532 nm lasers. See the text for the rationale. The EM-CCD camera continuously operated at a frame rate of 40 Hz. (c) Representative images and schematic illustrations of the possible entities being imaged (I–IV) using the alternating excitation laser sequence and two channel detection scheme.

Having demonstrated that these ZnO NC donors participate in EnT with freely diffusing A555 acceptors in solution, we deposited the NC donors and A555 acceptors on a quartz substrate and studied their emission behaviors using single molecule fluorescence microscopy. In a typical experiment, ZnO NCs and A555 solutions were sequentially spin coated onto a clean quartz slide and mounted on the stage of an inverted optical microscope equipped with a prism-type TIRF setup as shown in Figure 2a. Two laser sources excited the sample. A 355 nm laser selectively excited the ZnO bandgap, while a 532 nm laser selectively excited A555 molecules. A high-numerical aperture 60×/NA 1.2 objective collected the photons emitted from the sample. Image splitting optics provided dual wavelength images for simultaneous monitoring of the “defect” and “dye” channels, as defined by the bandpass filters shown in Figure 2b.

Figure 2b shows the pulse sequence for both laser sources. Each laser illuminates the sample for 1 s with a 0.5 s dark time between each pulse. This alternating excitation sequence starts with the 532 nm laser pulse to first image all A555 molecules in the field of view. Then, the first 355 nm laser pulse excites all ZnO NCs and should presumably induce defect-mediated EnT for some NC/dye pairs if an A555 molecule is located near the defect site, specifically a distance shorter than *R*_0_ = 3.1 nm according to our previous work.^38^ The rationale for alternating the excitation sequence is to track the dye emission as a function of increasing UV exposure time, which could cause unwanted photobleaching or photocatalytic oxidation of the dye via hole transfer from the NC to the dye. Hence, this excitation/detection scheme allows us to independently excite and detect photons emitted from ZnO NCs and A555 molecules. The wide field imaging approach enables us to simultaneously monitor hundreds of individual NCs and molecules, providing many isolated NCs and dye molecules that serve as valuable internal control data to potentially distinguish from the much smaller number of NC/dye pairs (defined in greater detail below). The EM-CCD camera was continuously operated with an exposure time of 25 ms (i.e., frame rate of 40 Hz). Figure 2c shows representative wide field fluorescence images for each excitation laser sequence and each detection channel. Dark-shaded rectangles denote the wavelengths covered by the bandpass filters in the image splitter as shown in Figure 1b. Upon excitation of the sample with the 532 nm laser, no signal appears in the ZnO defect channel (Figure 2c-I) because the 2.33 eV excitation photons do not have sufficient energy to excite the ZnO bandgap (3.4 eV). However, many individual fluorescent spots appear in the dye channel (Figure 2c-II). The fluorescent spots in the dye channel could represent non-specifically bound molecules adsorbed on the quartz slide as well as specifically bound dye molecules adsorbed on NCs, as schematically shown in Figure 2c-II.

Upon excitation of the same sample region with the 355 nm laser (bottom row in Figure 2c), bright spots appear in the dye and defect channels. The emission intensity in the defect channel (Figure 2c-III) can represent only ZnO NCs. To distinguish single NCs from NC clusters (Figure S6), we analyzed the intensities of all bright spots in the defect channel under 355 nm excitation and defined the large population or low-intensity objects as single NCs (Scheme S1 and Figure S7). On the contrary, the intensity of bright spots in the dye channel could stem from photoexcited ZnO NCs alone or NC/dye pairs (Figure 2c-IV) because the dye emission and ZnO defect PL signals overlap, as shown in panels b and c of Figure 1. One strategy to distinguish ZnO NCs alone from the ZnO/dye pair is to co-localize dye molecules observed in the dye channel under 532 nm excitation (red boxes in Figure 2c-II) with single ZnO NCs observed in the defect channel under 355 nm excitation (red boxes in Figure 2c-III).

### Super-resolution Imaging Analysis of ZnO/NC Pairs

We developed the following algorithm to identify single dye molecules “specifically bound” to single ZnO NCs. We define “specifically bound” NC/dye pairs as those entities whose individual donor and acceptor centroid positions are separated by a distance smaller than our localization precision (40 nm).^55,56^ To do so, we first classified the bright spots in Figure 2c-III as single ZnO NCs using an intensity thresholding procedure shown in Figure S7. Next, we segmented all single ZnO NCs into two populations: (1) 789 isolated ZnO NCs and (2) 78 ZnO NCs with A555 molecules located within the same diffraction-limited region of interest (ROI) in the image (i.e., a dye molecule and a single NC appear in the red boxes of Figure 2c). Figure S8 describes the procedure for identifying an A555 dye in the same ROI as an NC. Super-resolution imaging analysis^56^ of the A555 dye acceptor under the first 532 nm excitation pulse and the ZnO NC donor under the first 355 nm excitation pulse revealed two different populations: non-specifically bound and specifically bound NC/dye pairs. Figure S9 depicts the localization procedure used in this study.

**Figure 3:**
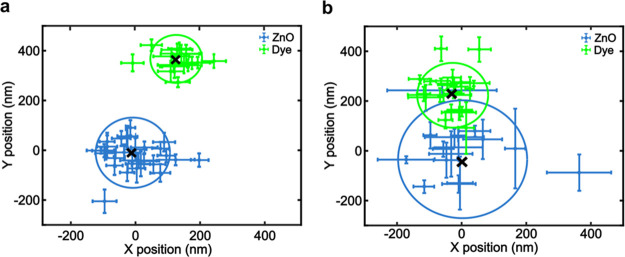
Super-resolution analysis of ZnO NC/A555 dye pairs separated by distances (a) larger and (b) smaller than our spatial resolution. The green and blue lines represent the coordinates and localization precision of A555 dyes and ZnO NCs for the first 1 s of 532 and 355 nm laser illumination, respectively. The black × points represent the average coordinates of all individual fits. The circles represent the error of the individual fits (two standard deviations). The ZnO centroid positions were determined from defect channel images under 355 nm excitation. The dye molecule positions were determined from dye channel images under 532 nm excitation.

Figure 3a shows a representative example of the non-specifically bound population (73 of 78 pairs), where the individual centroid positions in every frame for 355 and 532 nm laser illumination are shown as blue and green crosses, respectively. The black data points represent the average coordinates of the individual centroid locations, and the large circles represent the error in the individual fits (two standard deviations). A majority of candidate NC/dye pairs are located in the same diffraction-limited ROI but are separated by a distance larger than our spatial resolution (Figure 3a). The second, smaller population (5 of 78) can be defined as a NC/dye pair separated by a distance smaller than our spatial resolution (Figure 3b). Next, we analyzed the fluorescence intensity trajectories of the five specifically bound NC/dye pairs and compared the results to those for the large population of isolated ZnO NCs and non-specifically bound ZnO/dye pairs; both serve as internal control data because the donor–acceptor distances are much larger than *R*_0_ = 3.1 nm.

### Single Molecule, Single NC-Level Trajectory Analysis

**Figure 4:**
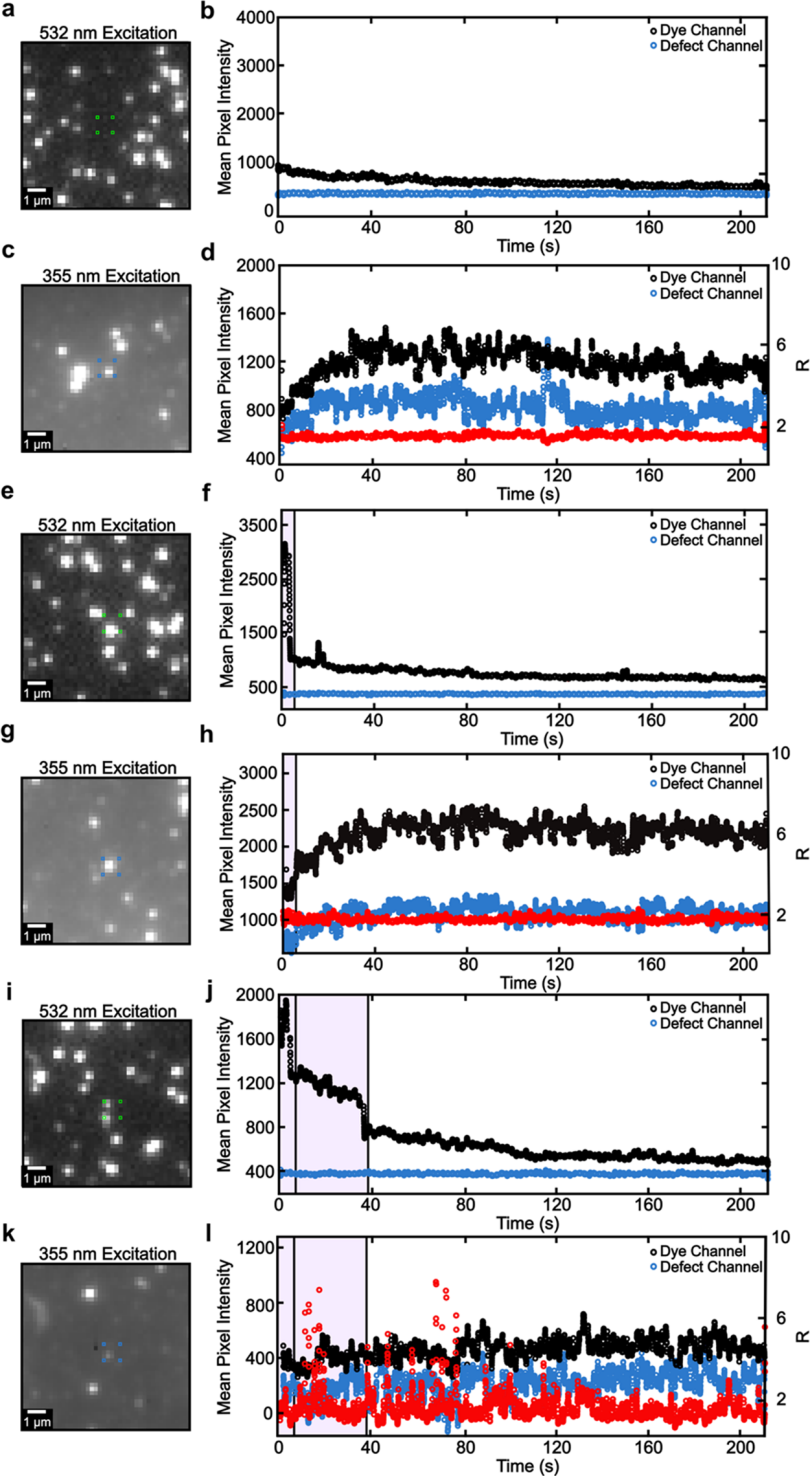
Trajectory analysis of (a–d) an isolated ZnO NC, (e–h) a non-specifically bound NC/dye pair, and (i–l) a specifically bound NC/dye pair. (a) Fluorescence image from the dye channel under 532 nm excitation. (b) Intensity trajectory from the ROI in panel a (green squares) in the dye (black trajectory) and defect (blue trajectory) channels. The pink shaded region highlights the time at which the dye molecule is “bright” under 532 nm excitation. (c) Fluorescence image from the defect channel under 355 nm excitation. (d) Same as panel b, but for 355 nm excitation. The red points show the dye/defect channel intensity ratio *R* (right axis). (e–h and i–l) Same as panels a–d but for non-specifically bound NC/dye and specifically bound NC/dye pairs, respectively.

Figure 4 shows representative fluorescence intensity trajectories for isolated ZnO NCs, a non-specifically bound NC/dye pair, and a specifically bound NC/dye pair. Panels a, e, and i of Figure 4 show representative fluorescence images from the dye channel during 532 nm excitation. The bright spots represent A555 molecules. Panels b, f, and j of Figure 4 show the fluorescence intensity trajectories of the ROIs in panels a, e, and i, respectively, of Figure 4 (green squares), where the black trajectory represents the intensity in the dye channel and the blue data points represent the intensity in the defect channel. Panels c, g, and k of Figure 4 show fluorescence images from the defect channel under 355 nm laser excitation. The bright spots represent ZnO NCs. Panels d, h, and l of Figure 4 show the trajectories in the dye and defect channels from the ZnO NC ROIs in panels c, g, and k, respectively, of Figure 4. The red trajectories represent the dye/defect channel intensity ratio, which will be discussed further in the next section.

Figure 4a–d shows an example of an isolated NC. The fluorescence image in Figure 4a shows no emissive objects inside the ROI of the dye channel during 532 nm excitation, but a clear bright spot appears in the same diffraction-limited volume in the defect channel under 355 nm excitation (Figure 4c). The fluorescence intensity trajectory in Figure 4b gradually decreases over time likely because the high-intensity laser excites emissive impurities in the solvent or substrate. The weak signal decays in ∼60 s, likely due to photobleaching of those impurities. The trajectories under 355 nm excitation show a slow intensity increase for the first 35 s (Figure 4d). The slow increase can be attributed to chemisorbed oxygen, which can initially quench the defect emission, which was experimentally observed through ensemble-level results by van Dijken et al.^57^

Figure 4e–h shows a representative example of a non-specifically bound NC/dye pair. A molecule appears in the same diffraction-limited volume as the NC (Figure 4e), and the fluorescence intensity in the dye channel during 532 nm excitation (Figure 4f, black trajectory) shows clear single step photobleaching behavior that is characteristic of a single molecule (pink shaded region); note the low intensity in the defect channel (Figure 4f, blue trajectory) because the A555 emission does not contribute intensity to the defect channel. Figure 4g shows a representative image of the single ZnO NC present in the same diffraction-limited location as the dye molecule in Figure 4e. Both defect and dye channel trajectories under 355 nm illumination show a slow intensity increase over the first 35 s of the movie (Figure 4h), similar to that of the isolated NC in Figure 4d.

Figure 4i–l shows a representative example of a specifically bound NC/dye pair. In this case, two dye molecules appear in Figure 4i, evidenced by two distinct photobleaching events in Figure 4j (shaded pink regions). We would have expected to observe simultaneous intensity bursts in the dye channel and intensity decreases in the defect channel under 355 nm excitation if EnT occurred. However, Figure 4l depicts only minor periods of anticorrelated intensity fluctuation behavior during the first 80 s of the movie. The intensity fluctuations, quantified in more detail below, do not appear in control data (Figure 4a–h). Unexpectedly, the fluctuations in Figure 4l extend beyond the photobleaching period of the second dye (second pink shaded region in Figure 4j). One possible cause of the intensity fluctuations is that an adsorbed “dark” dye molecule remains on the NC surface and provides a non-emissive pathway for photogenerated carriers in the NC, resulting in less defect emission from the NC. Figure S10 depicts photobleaching times for A555 dye acceptors, showing that “bright” dye molecules can exist for at least 60 s under these illumination conditions. Additionally, adsorbed molecular species such as O_2_ and ligands are known to quench NC emission.^57^

Regardless of the exact origin of the fluctuations, we performed further quantitative analysis to identify an EnT signature in these specifically bound NC/dye pairs that do not show clear anticorrelated FRET behavior. To do so, we calculated the dye/defect channel intensity ratio (*R* = *I*_dye_/*I*_defect_) for trajectories under 355 nm excitation. We would expect large *R* values to indicate EnT occurs because the process should experimentally manifest as an increase in the dye channel intensity and a simultaneous decrease in the defect channel intensity (see ensemble-level results in Figure 1c). The *R* values remain constant in control experiments (Figure 4d,h). However, distinct *R* intensity fluctuations appear in the specifically bound case (Figure 4l).

### Single Molecule, Single NC-Level Channel Ratio Analysis

Next, we quantitatively compared the intensity trajectories of isolated NCs to those of specifically bound NC/dye pairs. Our hypothesis is that EnT is most likely to manifest as intensity fluctuations in the beginning of the trajectory. To test this hypothesis, we calculated a single *R* value for each NC, *R̅*, by dividing the average *R* of the first 30 frames by the average *R* of the last 30 frames. If EnT occurs in the specifically bound NC/dye pairs, we would expect to observe larger *R̅* values in the specifically bound cases because the dye channel intensity should increase more and the defect channel intensity should decrease more during the beginning of the experiment than at the end of the experiment.

**Figure 5:**
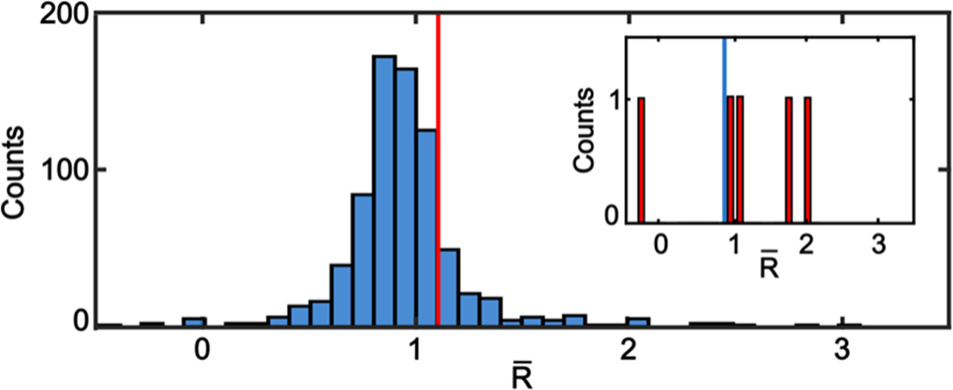
Distribution of *R̅* values for 789 isolated NCs (blue) vs the average value of five specifically bound ZnO/dye pairs (vertical red line). *R̅* values represent the average *R* = *I*_dye_/*I*_defect_ under 355 nm excitation of the first 30 frames divided by the average *R* of the last 30 frames. The inset shows the individual values of the five specifically bound pairs. The blue vertical line is the average value of the isolated NCs.

Figure 5 shows the distribution of *R̅* values for 789 isolated NCs versus the average of five specifically bound NC/dye pairs. The average *R̅* value of the isolated NCs is 0.92. We would have expected this value to equal 1.0 if the defect emission of the ZnO NCs was stable with illumination time. However, we observe that the low-energy portion of the broad defect PL peak (dye channel) increases in some NCs (large *R̅* values in the distribution) and decreases in others (low *R̅* values). This phenomenon could be due to an evolution of optically active emissive defects in ZnO (e.g., zinc interstitials vs oxygen vacancies) under these illumination conditions, as observed previously.^58−72^ Despite the complex defect emission photophysics of these ZnO NCs,^66,73−75^ we consistently observe larger *R̅* values for the specifically bound pairs than for the isolated ensemble average (Figure 5, inset). In some cases, we observe negative *R̅* values because the background ROI intensity can exceed the NC ROI intensity during the first frame. The calculated *p* value for the data in Figure 5 is 0.65. One explanation for the large *p* value is the significantly different sample size (789 vs 5). Amassing experimental data from a larger population of specifically bound NC/dye pairs could provide further evidence that the photophysics of ZnO NCs alone differ from those of specifically bound NC/dye pairs. In summary, Figure 5 shows a quantitative difference between isolated NCs and specifically bound NC/dye pairs that is consistent with the ensemble-level results in Figure 1c (i.e., dye channel enhancement and defect channel quenching). The Discussion focuses on possible reasons why we did not observe clear anticorrelated FRET behavior under these experimental conditions.

## Discussion

Here we ask the critical question: Why do the trajectories of specifically bound NC/dye pairs not show clear anticorrelated FRET intensity fluctuations even though ensemble-level studies show clear FRET behavior? One possibility is that the separation distance of the NC/dye pairs in these “specifically bound” pairs is larger than *R*_0_ (3.1 nm). Our lab is pursuing the synthesis of ZnO/dye conjugates, in which a silane layer encapsulated dye molecules around the NC core, on the basis of a previously published synthesis.^33^ Chemically linking the NC and dye is likely necessary to study a statistically meaningful population of specifically bound pairs because our Monte Carlo simulations (Figure S11) suggest that random co-localization of the NCs and dyes produces at least one non-specifically bound NC/dye pair in 50% of deposition experiments. These physically attached NC/dye constructs will allow us to rule out the possibility that the minor intensity fluctuations in Figure 4l are not a consequence of the spin coating method producing “specifically bound” pairs with a separation distance slightly larger than *R*_0_.

Another possibility is that the defect transition dipole moment of the NC donor does not overlap with the dye acceptor.^76−80^ The ensemble-level measurements permit freely diffusing dye molecules to approach and/or temporarily adsorb to all surfaces of the ZnO NCs. This random attachment likely allows for effective dipole–dipole coupling between NC donors and dye acceptors.^2^ On the contrary, both the orientation factor and the separation distance of the donor and acceptor are “locked” in the single molecule imaging experiments. It is possible that the dipole moment of the defect donor site does not overlap with the single surface-adsorbed molecule. It is also possible that the dye molecule binds to the opposite side of the 4.5 nm diameter NC where the defect site is located, causing the donor–acceptor distance to exceed 3.1 nm. Synthesizing and imaging the silane-encapsulated dye/NC conjugates mentioned above with polarization-dependent illumination conditions should allow us to quantify a larger number of NC/dye orientations with minimal separation distance.

**Figure 6:**
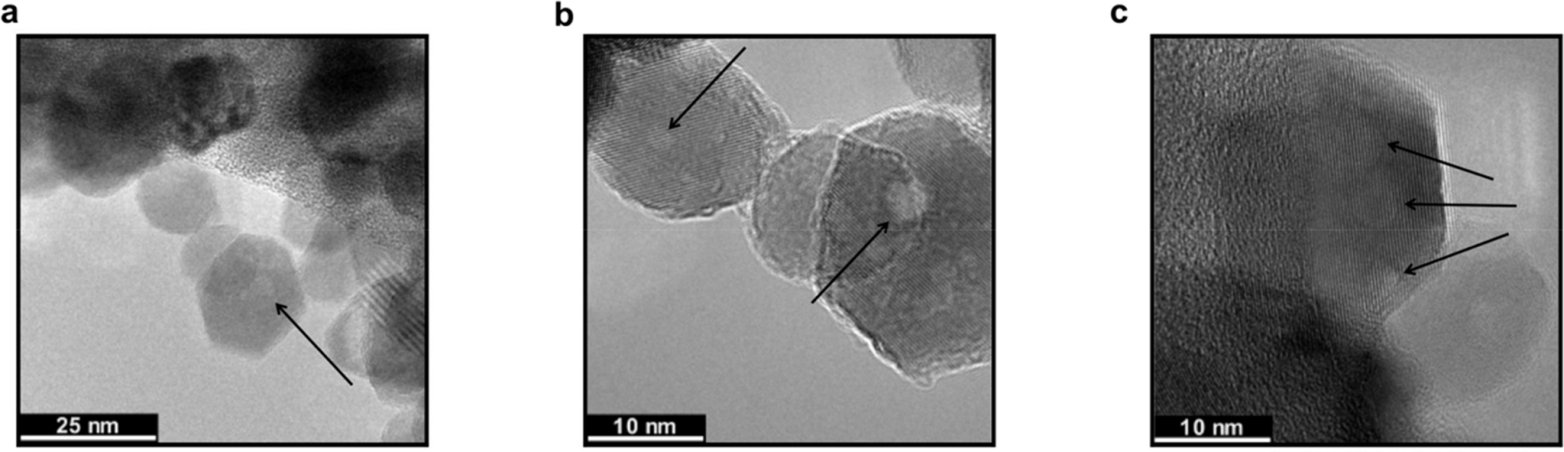
TEM images of ZnO NCs with various defective regions highlighted by the black arrows. Note that panels a and b show the same NCs at different magnifications. (c) Single ZnO NC showing multiple defective regions.

Another explanation for the lack of defect emission quenching is the fact that multiple defect sites could contribute to the emission signal. In this scenario, multiple emitters dominate the emission signal and the single defect site participating in energy transfer cannot be observed. High-resolution TEM imaging of our NC sample (Figure 6) revealed defective voids that could be associated with multiple lattice defect sites.^68,69,81−86^

**Figure 7:**
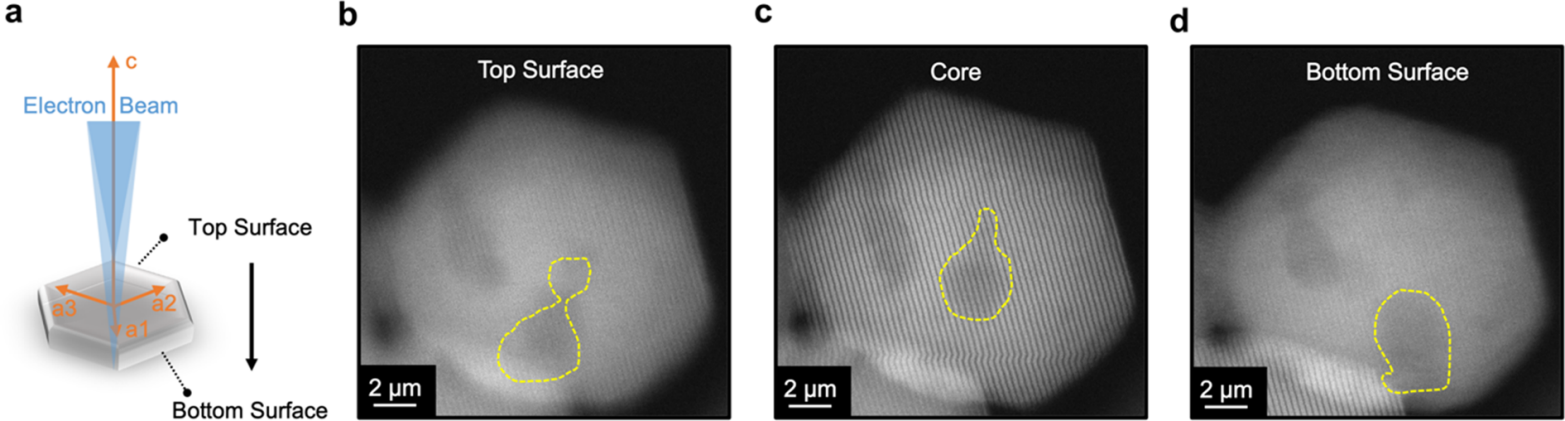
STEM images of a single nanocrystal acquired with different defocus values. (a) Cartoon illustration of the imaging scheme, where STEM images are acquired as a function of *z* focus position. STEM images of the (b) top surface, (c) core, and (d) bottom surface of the NC. Yellow dashed lines show different shapes of multiple defective regions.

Additionally, we recorded a focal series of cross-sectional STEM images to understand the distribution of porous defects in three dimensions. Figure 7 shows the images selected from the STEM focal series, which revealed that porous defects exist within the NC core and at the surface. Multiple nanometer-scale voids of various shapes appear in a single particle as the focus position changes. The theoretical description of the donor dipole moment and its orientation relative to the acceptor dipole remains unclear for these large crystallographic defects. We also calculated the number of defects per NC on the basis of literature values of O and Zn vacancy concentrations (Table S1). We concluded that there are, on average, 0.5 defect per NC, in addition to the large defective voids in Figure 6. In summary, these NCs likely have more than one defect site per NC, and therefore, a single dye molecule may not fully quench the defect emission from the sample.

## Conclusion

We studied defect-mediated EnT between ZnO NC donors and A555 dye acceptors using ensemble-level fluorescence spectroscopy and single molecule-level fluorescence microscopy. The ensemble-level experiments show clear FRET behavior, where the dye acceptor emission enhances and the NC donor emission quenches. Spin coating the dye on NC-coated quartz slide samples produced 73 NC/dye pairs in the same diffraction-limited space and five specifically bound NC/dye pairs. We observed minor fluorescence intensity fluctuations in the trajectories of specifically bound pairs that did not appear in the trajectories of isolated NCs. Large separation distances and poor dipole–dipole coupling could explain why clear anticorrelated donor/acceptor intensity fluctuations do not appear in the intensity trajectories under these experimental conditions.

## Data Availability

The data that support the findings of this study are available from the corresponding author upon reasonable request.

## Supporting Information Available

Additional synthesis information, single molecule imaging, image analysis procedures, and Monte Carlo simulations (PDF)
