# Supplementary material for: Toward Imaging Defect-Mediated Energy Transfer between Single Nanocrystal Donors and Single Molecule Acceptors
Source: Chem Biomed Imaging. 2023 Apr 4;1(2):168–78. doi: 10.1021/cbmi.3c00015 (PMC11504506; doi:10.1021/cbmi.3c00015)
Supplement: Supplementary file 1 — im3c00015_si_001.pdf [file im3c00015_si_001.pdf]

Supporting Information For

Towards Imaging Defect-Mediated Energy Transfer Between Single Nanocrystal Donors and Single Molecule Acceptors

Danielle R. Lustig[1,†] Zach N. Nilsson[1,†] Justin T. Mulvey[3,5], Wenjie Zang[5], Xiaoqing Pan[5,6,7], Joseph P. Patterson[3,4,5], and Justin B. Sambur[1,2]*

[1]Department of Chemistry, Colorado State University, Fort Collins, CO 80523-1872, USA

[2]School of Advanced Materials Discovery, Colorado State University, Fort Collins, CO 80523-1872, USA

[3]Center for Complex and Active Materials, University of California, Irvine, Irvine, CA 92697-2025, USA

[4]Department of Chemistry, University of California, Irvine, Irvine, California 92697-2025, USA

[5]Department of Materials Science and Engineering, University of California, Irvine, Irvine, CA 92697-2025, USA

[6]Irvine Materials Research Institute, University of California, Irvine, Irvine, CA, 92697-2025, USA

[7]Department of Physics and Astronomy, University of California, Irvine, Irvine, 92697-2025, CA, USA

Correspondence to *jsambur@colostate.edu

[†]equal contribution

1. Sample Preparation

ZnO NCs were synthesized following the procedure in our previous publication with the only modification being that a single aliquot was taken after 6 hours reaction time. This method yielded 4.5 nm-diameter particles based on the size series depicted in reference S1.[1]

ZnO NC-coated quartz slides were typically prepared by spin coating 70-150 nM solutions of ZnO NCs onto a cleaned quartz slide at 1000 RPM. A 0.1 nM ethanolic solution of A555 dye was drop-cast directly on top of the NCs. The droplet of dye was allowed to stand on the slide for one minute before being completely removed by brisk nitrogen stream. The quartz slides were pre-cleaned by immersion in a 3:1 solution of sulfuric acid and hydrogen peroxide (Piranha solution) for at least 12 hours. Slides were then rinsed with 10 MΩ water and stored in spectrophotometric grade ethanol until use.

Immediately prior to placing the sample on the microscope, a #1 glass coverslip was placed onto the sample side of the slide, sandwiching a drop of Immersol W 2010 immersion oil (Zeiss) between the slide and coverslip. The immersion oil solvent kept the NCs and dyes adsorbed on the quartz slide because neither object was soluble in that solvent. We also observed that the NC emission was more stable in Immersol media than in air, presumably because O_2 quenches the ZnO defect emission.[2]

2. Imaging Setup and Data Acquisition

Fluorescence images were acquired using the total internal reflection (TIR) fluorescence setup pictured in Figure 2 of the main text. The sample was placed onto an Olympus IX73 inverted microscope with the coverslip side facing down and brought into optical focus using transmitted white light. Next, a small drop of index matching oil was placed onto the top surface of the slide and a quartz prism was placed on top of the oil and held in place so that the sample slide could move freely under the prism while maintaining good contact. 355 and 532 nm laser light was directed into the prism at the critical angle for TIR. The evanescent excitation field excited NCs and molecules on the slide surface while limiting the penetration of the field into the bulk solution, reducing background signal.

Illumination was provided by two laser sources: a 355 nm laser (Coherent Genesis CX) selectively excites the ZnO NCs while the 532 nm laser (Coherent Obis) selectively excites the A555 molecules. Photons emitted from the sample were collected through a 60× microscope objective (NA = 1.2, Olympus, UPLANSAPO60x/W) before passing through a long pass filter

(cutoff wavelength: 440 nm). Then the photons enter the image splitter (Hamamatsu Gemini W-view) which uses a dichroic mirror (cutoff wavelength: 550 nm) to split the image based on wavelength. One image is the "dye channel" because the photon stream passes through a 585/40 bandpass filter. The second bandpass filter (490/60) was chosen to pass the ZnO defect emission and exclude any photons from A555, forming the "defect channel".

The two channels were projected onto an EMCCD camera (Andor iXon 897) to produce a single image, split vertically (Figure S1). The split images allow for the behavior of the NCs to be observed without interference from A555 by using the defect channel while also allowing for observations of the A555 in the dye channel. Data was acquired as a stream of fluorescence images from the camera, forming a "movie". Movies were analyzed to determine the behavior of the ZnO and A555 separately after they had been mixed following the sample preparation procedure outlined above. Because the ZnO defect emission is so broad, the NC emission should appear in both channels, making the NCs easy to distinguish from non-NC objects.

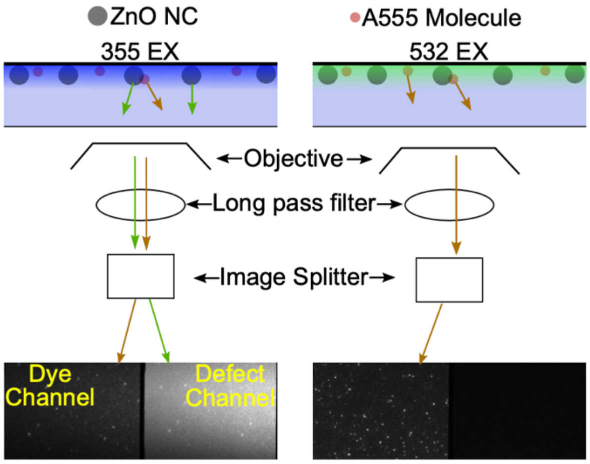

Figure S1. Cartoon depiction of the prism-type total internal reflection fluorescence microscopy setup.

3. Image Processing

Segmenting movies by 355 nm and 532 nm laser illumination.

In a typical experiment both laser sources are used to observe the locations of A555 molecules using 532 nm excitation and the behavior of ZnO NCs with 355 nm excitation. The sample is exposed to each laser in one second pulses with half a second of dark time between each

pulse. Alternating the excitation wavelength allows for A555 molecules to be observed independently of the NCs throughout the entire movie. The movies can then segmented according to the illumination condition (355 or 532 nm excitation) and detection channel (dye or defect).

We used MATLAB to segment the images of an entire movie according to the illumination condition. To do so, we calculated the average frame intensity trajectory from all pixels of every image shown in Figure S1a-bottom left, creating the average pixel intensity verses time plot shown in Figure S2a. Figure S2b shows a zoom-in view of the first 60 s of the experiment. Here, the average pixel intensity (black line) shows distinct square-wave-like features due to the alternating excitation source. The two lasers create two distinct intensity levels visible in Figure S2b. The 355 nm laser produces higher intensity peaks than the 532 nm excitation laser. We determine the laser on/off points by taking the derivative of the trajectory in Figure S2a and determining the distinct positive and negative derivative transitions using blue and green triangles (the results are shown in Figure S2b).

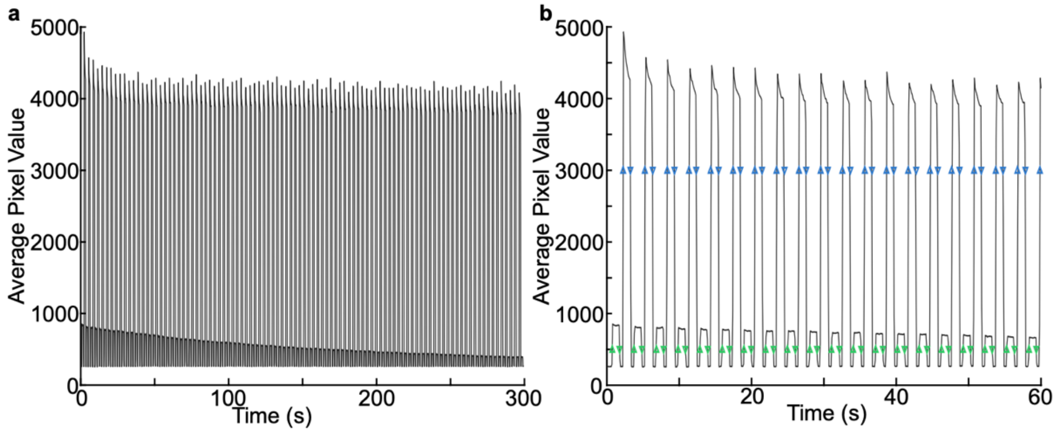

Figure S2. a) Representative average pixel value versus time trajectories from all images in the movie with 0.1 nM A555 deposited onto ZnO NCs. **b**) The same trajectory as shown in a) but with a shortened time axis to better show the data. Blue triangles in b) mark the frames where the 355 nm laser turns on and off. The green triangles mark the frames where the 532 nm laser turns on and off.

<u>*Selecting regions of interest (ROIs) and transforming ROIs from the defect channel to the dye channel.*</u>

Having segmented the movies by illumination condition, we selected ROIs in the defect channel and transformed them to the dye channel to ensure our intensity trajectory calculations of NCs and dyes under 355 nm and 532 nm illumination originated from the same locations in the

image. This step was necessary because NCs do not appear in the dye channel under 532 nm illumination and dye molecules do not appear in either channel under 355 nm illumination. To do so, we computed a single average image from all frames under 355 nm illumination (Figure S3a). We clicked the centroid position of each bright object in the defect channel during 355 nm excitation and created a 4×4 pixel box centered around each bright spot (a subset of ROIs are shown in Figure S3b-left). Those ROIs will be referred to as the NC ROIs to distinguish them from dye ROIs. This procedure yielded 1,078 NC ROIs (see Scheme S1 below). We also created background NC ROIs of equal size and adjacent to the NC ROIs to create background subtracted PL intensity trajectories (see Figure S4).

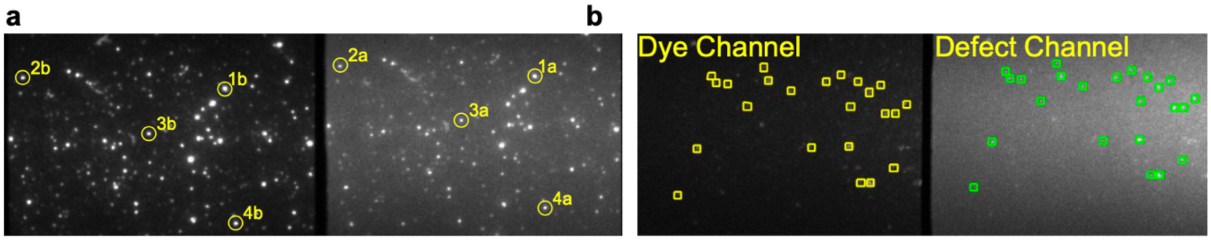

Figure S3. Fluorescence images of ZnO NCs under 6mW 355 nm excitation projected through the imagesplitter. The yellow circles in a) denote the spots that were used to calculate the transformation matrix. b) Results of the transformation process, where the ROIs in the defect channel (green boxes) were transformed to the dye channel (yellow boxes). Background ROIs are omitted from this image for clarity.

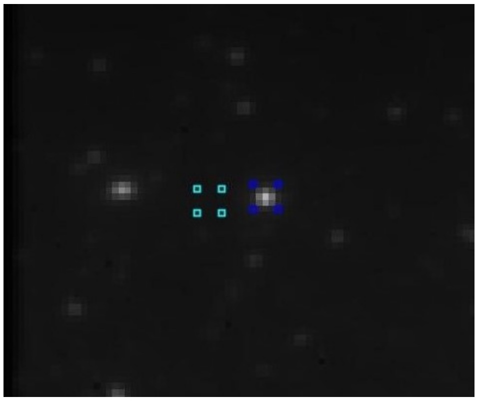

Figure S4. Cropped fluorescence image showing NC ROIs (blue squares define the corners) and background NC ROIs (cyan squares).

After creating the ROIs in the ZnO defect channel, they were transformed to the dye channel using a geometric transformation matrix. The matrix was created by selecting sets of matching

coordinates in both images; ZnO NCs appear in both channels under 355 nm laser illumination (Figure S3a). These sets of points were passed to the MATLAB function "fitgeotrans", which outputs a geometric transformation matrix. The transformation matrix is then applied to the vertices of the ROIs created in the defect channel, creating a new set of vertices which have been translated onto the dye channel, as shown in Figure S3b. This transformation is applied to both the NC ROIs and the background ROIs. We estimate the error in the overlay procedure is 25 nm.[3] After selecting the ROIs, the average pixel intensity in each ROI of each frame was calculated for both channels. This forms an intensity versus time trajectory for any ROI, as shown in the main text.

Dye ROIs were created by averaging frames in the dye channel during 532 nm excitation (Figure S5). We clicked the centroid position of each bright object in the dye channel during 532 nm excitation and created a 4×4 pixel box centered around each bright spot.

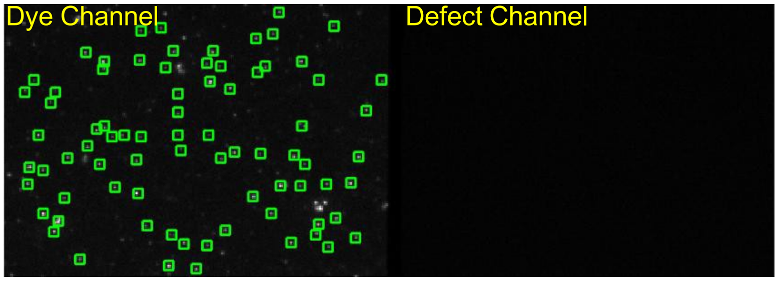

Figure S5. Fluorescence image of A555 molecules under 6mW 532 nm excitation. Green boxes represent the vertices of the dye ROIs.

Distinguishing Single NCs from NC clusters.

The ZnO NC coverage on the quartz substrate is heterogeneous, containing both single ZnO NCs and NC clusters. For example, Figure S6 shows a florescence image of ZnO NCs under 355 nm excitation. The yellow arrows indicate two objects in each channel that are much larger and brighter than most of the other objects in the image. These large, bright spots can likely be attributed to NC clusters. We developed the following image analysis algorithm, schematically shown in Scheme S1, to quantitatively distinguish single NCs from NC clusters.

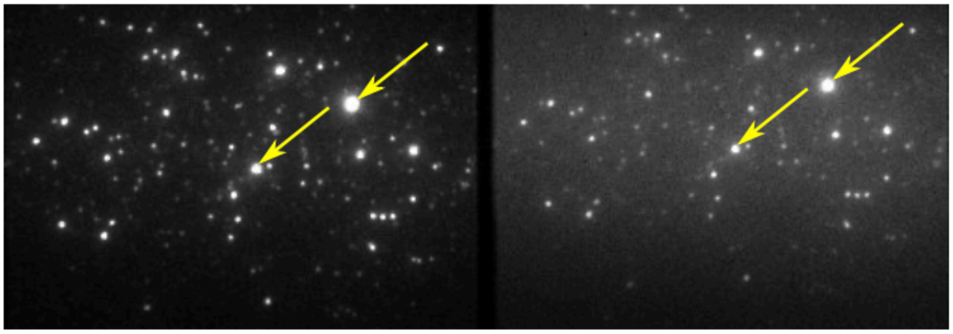

Figure S6. Wide field florescence image of ZnO nanocrystals. Yellow arrows point to objects that are considerably larger and brighter than the majority of other objects (clusters).

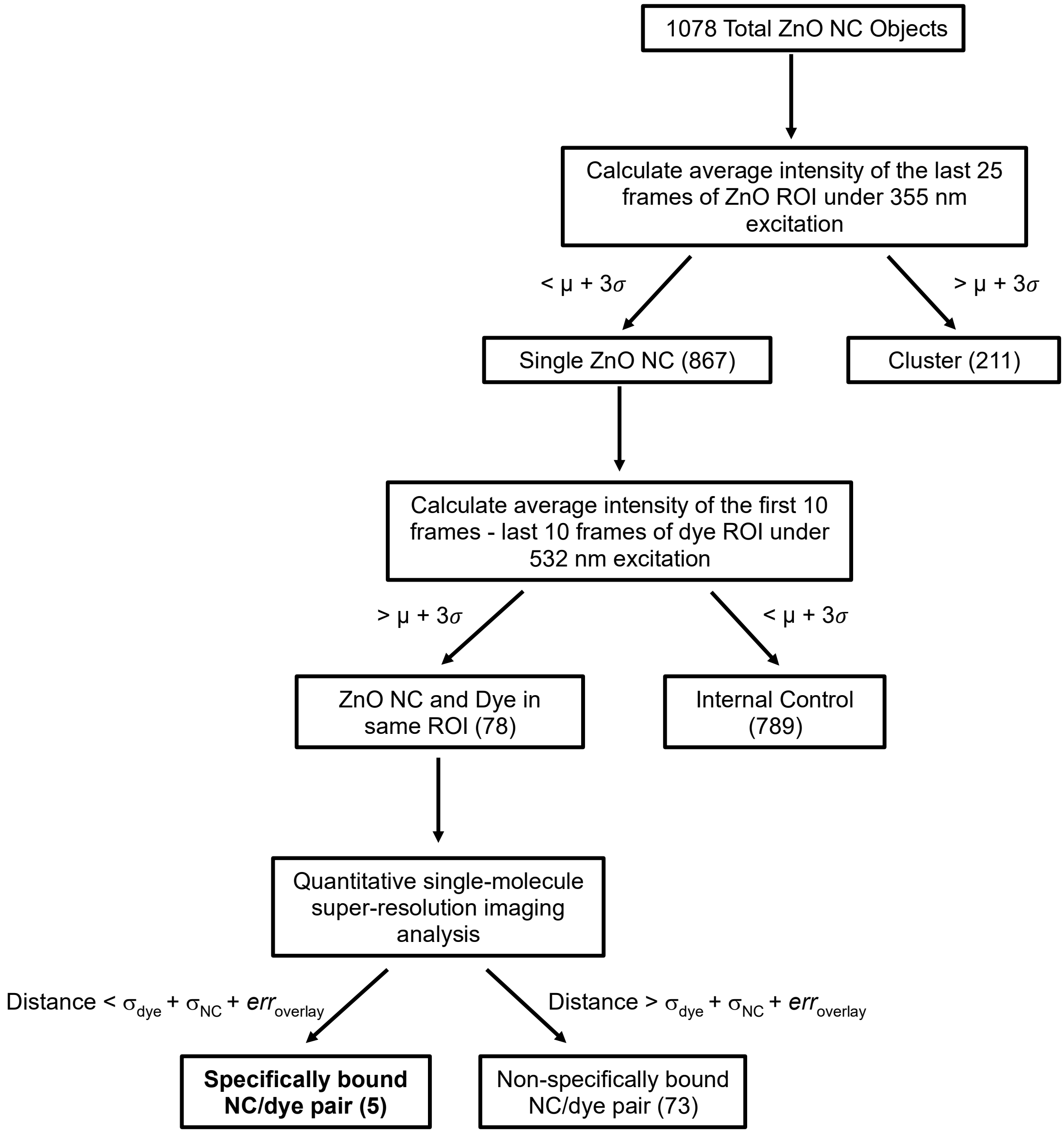

Scheme S1. Flow chart outlining the image analysis algorithm and filtering steps to quantify single dye molecules, single NCs, and specifically and non-specifically bound NC/dye pairs. "Th" represents threshold value. The numbers in parenthesis represent the number of filtered entities after each step.

To separate single NCs from NC clusters, we calculated the intensity for all 1,078 NC ROIs. Figure S7 shows a representative histogram for one of our movies of the average intensity values from the last 25 frames from all ROIs in the defect channel during 355 nm excitation. We assign the large population at low intensity to single NCs and the high intensity objects to clusters. We defined an intensity threshold for single NCs by fitting the large population with a single

component Gaussian function to establish the mean (μ) and standard deviation (σ) of the single particle population. The intensity threshold (Th in Scheme S1) was defined as three times the standard deviation above the mean (μ+3σ). The vertical grey line in Figure S7 serves as the intensity threshold for single ZnO NCs, meaning any NC whose intensity is less than this amount is defined as a single ZnO NC. We repeat this procedure for each movie.

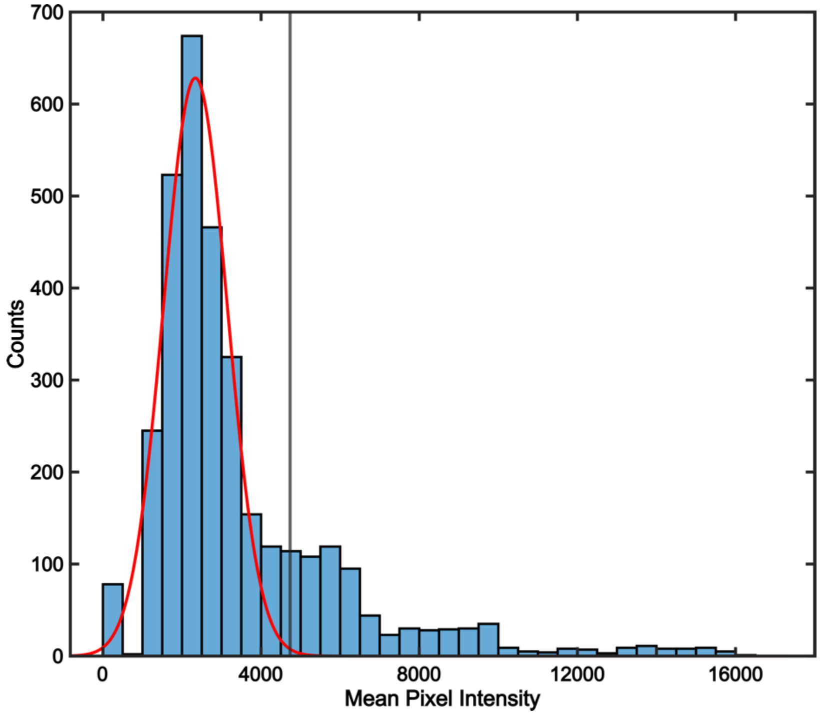

Figure S7. Histogram of average PL emission intensity of all bright objects in the defect channel under 355 nm excitation. The red line is a Gaussian fit to the large population at low intensity that can be assigned to single ZnO NCs. The vertical line represents the mean plus 3 standard deviations of the Gaussian fit result. That vertical line serves as the intensity threshold for single ZnO NCs, meaning any NC whose intensity is less than this amount is defined as a single ZnO NC.

Do single NCs also have a dye molecule located in the same diffraction-limited volume?

To determine which NCs do and do not have a dye molecule present in the same ROI, we examined the intensity trajectory from an NC ROI *in the dye channel during 532 nm excitation*. Figure S10a shows a representative trajectory of a dye molecules observed in the dye channel under 532 nm excitation. To determine whether a dye molecule was also present in the same NC ROI, we calculated the average intensity of the first 10 frames and subtracted that value from the average intensity of the last 10 frames. Figure S8 shows a representative histogram of those results for one movie and was repeated for all 867 single NCs. We observed a large, low intensity

population that can be attributed to "empty" ROIs. The high intensity population can be attributed to dye molecules that are initially present in the ROI. Fluorescence intensity trajectories of the diffraction-limited spots show single-step photobleaching (Figure S10), further confirming our assignment of these spots as single molecules. We fit the histogram with a Gaussian function to establish a mean and standard deviation for the low intensity "empty" population. We defined an intensity threshold as three times the standard deviation above the mean ($\mu + 3\sigma$). Any objects greater than this threshold (gray line in Figure S8) are defined as dye molecules in a NC ROI. In doing so, we identify all single NCs with a dye molecule located in the same diffraction-limited space.

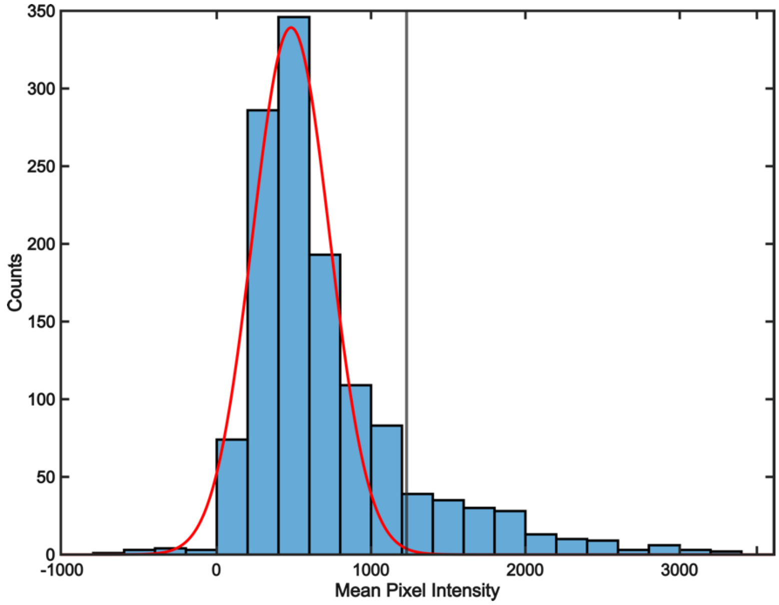

Figure S8. Histogram of NC ROI intensities located in the dye channel under 532 nm excitation. The intensities represent the average intensity of the last 10 frames subtracted from the average intensity of the first 10 frames. The red line is a Gaussian fit to the large population at low intensity that can be assigned to non-dye objects present in the ROI. The vertical line represents the mean plus 3 standard deviations of the Gaussian fit result. The vertical line serves as the intensity threshold for A555 dyes, meaning any ROI whose intensity is greater than this amount is defined as a dye.

<u>Distinguishing specifically bound from non-specifically-bound NC/dye pairs.</u>

The details of the super-resolution imaging analysis and MATLAB code are provided in the Supplementary Information of Sambur *et al*[3]. Super-resolution analysis fits were performed for the 78 candidate ROIs that contain a single NC and a dye molecule. Each candidate spot in each frame was fit with a 2D Gaussian function during the first 1 s under both 355 and 532 nm excitation to

determine the centroid position of the NC and dye, respectively. We obtain the centroid positions of the dye and NC during the first 1 s of the experiment (see blue and green data points in Figure 3 of the main text). We calculated the localization error according to Eq. 6 in Mortensen *et al.*[4] and plotted those values as error bars on the blue and green data points in Figure 3 of the main text. Figure S9 shows the distribution of localization error values in both x and y dimensions for NC ROIs under 355 nm illumination. The average localization error is 39.7 nm.

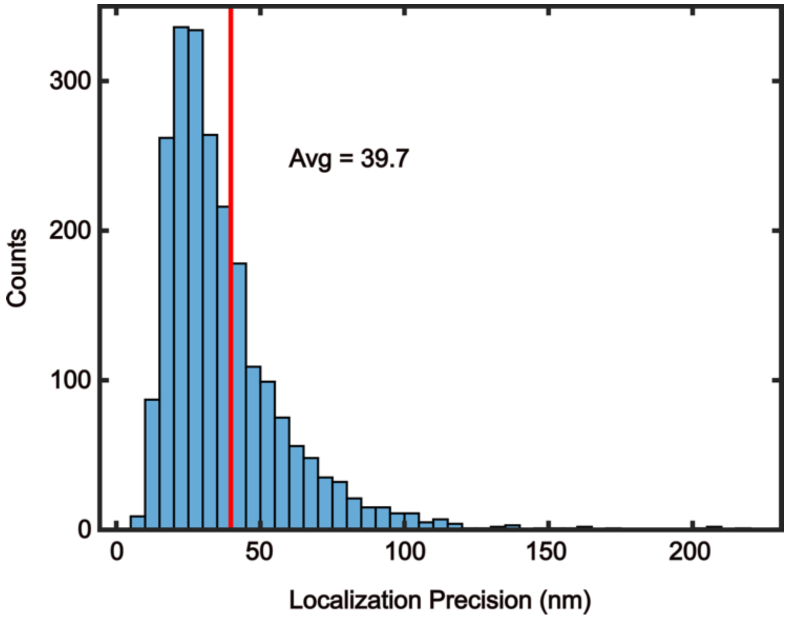

Figure S9. Histogram of the localization error in both the x and y dimensions for ZnO NC ROIs illuminated with 355 nm light. The values were calculated according to ref. S4. The gray bar represents the average localization error (39.7 nm).

To distinguish specifically bound versus non-specifically bound NC-dye pairs, we first assumed the NC and dye do not move during the acquisition time and, therefore, calculated the average x and y coordinates of the NC and dye to determine their positions with super-optical resolution (large black data points in Figure 3 of the main text). We also calculated the standard deviation (σ) of the centroid positions of the dye and NC from all the individual frames. σ_{dye} and σ_{NC} represents the error in the mean position of the dye and NC, respectively (indicated by a circle with radius μ in Figure 3 of the main text). Note, the error in the overlay procedure ($err_{overlay}$) is approximately 25 nm.[3] If the separation distance between the NC and dye were less than (σ_{dye} + σ_{NC} +$err_{overlay}$), then we defined the NC-dye pair as specifically bound.

4. Dye Photobleaching Behavior

Fluorescence intensity trajectories of single dye molecules exhibited abrupt photobleaching. Figure S10 shows a trajectory from an ROI in the dye channel under 532 nm excitation. Only the intensity values recorded during 532 nm excitation are shown. Two sharp intensity drops are observed, one at early times and another at 125 seconds. These intensity drops are likely caused by sequential photobleaching events of two different molecules. Figure S10b shows a histogram of these photobleaching events for 296 dye molecules. The average photobleach time was 53 seconds (red line), which is long enough to observe intensity fluctuations in the defect channel under 355 nm excitation.

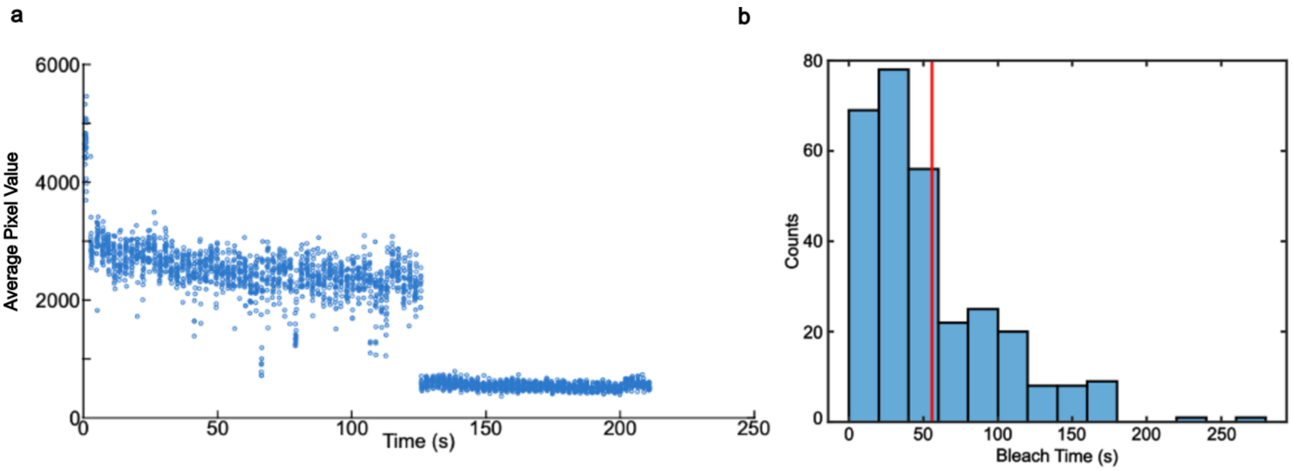

Figure S10. Intensity trajectory from a single dye ROI under 532 nm excitation. a) intensity trajectory from a ROI created in the dye channel. Each blue point represents the average intensity inside the ROI during one frame of the movie. b) Histogram of photobleach times for 296 A555 dye molecules. The red line displays the average photobleach time of 53 seconds.

5. Estimating the number of defect sites per NC

Table S1 displays calculations of defects/NC based on previously reported defect concentrations. To calculate defects/NC, it was assumed that the NCs were a sphere, and the NC volume was calculated from the TEM data in Figure 1a of the main text.

Table S1. Calculated defects/NC based on previously reported defect concentrations.

Defect Type	Sample	Experimental Conditions	Method	Defect Concentration (cm^{-1})	Defects/NC	Source
Oxygen Vacancies	72-atom supercells of wurtzite ZnO	High temperature equilibrium grown ZnO	First-principles calculations	1×10^{17}	0.0048	Lany and Zunger
Oxygen Vacancies	Bulk ZnO	700 °C	Hybrid density functionals	1×10^{19}	0.48	Clark *et al.*
Zn vacancies	Undoped bulk ZnO crystals grown by the seeded vapor phase	10–300 K	Positron annihilation measurements	2×10^{15}	9.5×10^{-4}	Saarinen *et al.*
Zinc interstitial	Simulated wurtzite ZnO	900 K	Local Density Approximations	1×10^{19}	0.48	Oba *et al.*
Oxygen vacancies and Zinc interstitials	Bulk ZnO crystals grown by the seeded chemical vapor transport	Annealed in zinc vapor at 1100 °C for 30 min	Smakula's equation	2×10^{18}	0.081	Halliburton *et al.*

6. Monte Carlo Simulations of NC-dye coverages

We performed Monte Carlo simulations to calculate how many non-specifically bound NC-dye pairs one would expect to observe based on random co-localization. We randomly placed 145 NCs and 244 dye molecules in a 44.4 µm × 64.9 µm area (corresponding to a 167 pixel × 244 pixel region of our image) and determined the number of occurrences where 1 NC and 1 dye were located within a 266 nm × 266 nm area (one square pixel). The NC and dye quantities correspond to the average number of NCs and dyes observed after 6 deposition experiments. We observed at least one non-specifically bound NC-dye pair in 5,808/10,000 simulations, and 8,664 total pairs (Figure S11). Therefore, one would expect to observe one non-specifically bound NC-dye pair in 58% of the deposition experiments. Experimentally, we observed 73 non-specifically bound NC-dye pairs in 6 deposition experiments. The higher number of experimental

observations is likely because the carboxylic acid group interacts with the oxide surface. The Monte Carlo simulations did not consider interactions between the NCs and dyes.

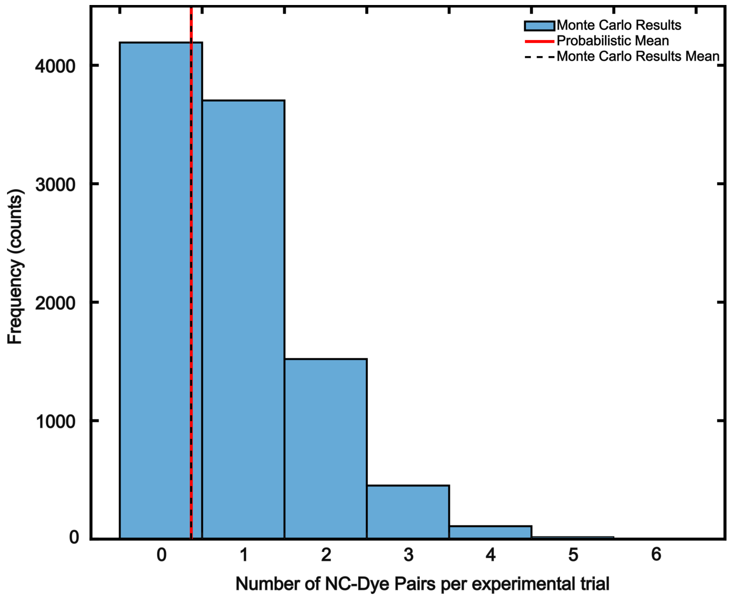

Figure S11. Distribution of the number of non-specifically bound NC-dye pairs observed after randomly placing 145 NCs and 244 dye molecules in a 44.4 μm × 64.9 μm area. The results represent the cumulative number of pairs observed after 10,000 Monte Carlo simulations.

7. References

(S1) Nilsson, Z. N.; Beck, L. M.; Sambur, J. B. Ensemble-Level Energy Transfer Measurements Can Reveal the Spatial Distribution of Defect Sites in Semiconductor Nanocrystals. *J. Chem. Phys.* **2021**, *154* (5), 054704. https://doi.org/10.1063/5.0034775.

(S2) van Dijken, A.; Meulenkamp, E. A.; Vanmaekelbergh, D.; Meijerink, A. Influence of Adsorbed Oxygen on the Emission Properties of Nanocrystalline ZnO Particles. *J. Phys. Chem. B* **2000**, *104* (18), 4355–4360. https://doi.org/10.1021/jp993998x.

(S3) Sambur, J. B.; Chen, T.-Y.; Choudhary, E.; Chen, G.; Nissen, E. J.; Thomas, E. M.; Zou, N.; Chen, P. Sub-Particle Reaction and Photocurrent Mapping to Optimize Catalyst-Modified Photoanodes. *Nature* **2016**, *530* (7588), 77–80. https://doi.org/10.1038/nature16534.

(S4) Mortensen, K. I.; Churchman, L. S.; Spudich, J. A.; Flyvbjerg, H. Optimized Location Analysis for Single- Molecule Tracking and Super-Resolution Microscopy. *Nat. Methods* **2010**, *7* (5), 377-381.

https://doi.org/10.1038/nmeth.1447.

(S5) Lany, S.; Zunger, A. Dopability, Intrinsic Conductivity, and Nonstoichiometry of Transparent Conducting Oxides. *Phys. Rev. Lett.* **2007**, *98* (4), 045501. https://doi.org/10.1103/PhysRevLett.98.045501.

(S6) Clark, S. J.; Robertson, J.; Lany, S.; Zunger, A. Intrinsic Defects in ZnO Calculated by Screened Exchange and Hybrid Density Functionals. *Phys. Rev. B* **2010**, *81* (11), 115311. https://doi.org/10.1103/PhysRevB.81.115311.

(S7) Saarinen, K.; Hautakangas, S.; Tuomisto, F. Dominant Intrinsic Acceptors in GaN and ZnO. *Phys. Scr.* **2006**, *T126*, 105–109. https://doi.org/10.1088/0031-8949/2006/T126/024.

(S8) Oba, F.; Choi, M.; Togo, A.; Tanaka, I. Point Defects in ZnO: An Approach from First Principles. *Sci. Technol. Adv. Mater.* **2011**, *12* (3), 034302. https://doi.org/10.1088/1468-6996/12/3/034302.

(S9) Halliburton, L. E.; Giles, N. C.; Garces, N. Y.; Luo, M.; Xu, C.; Bai, L.; Boatner, L. A. Production of Native Donors in ZnO by Annealing at High Temperature in Zn Vapor. *Appl. Phys. Lett.* **2005**, *87* (17), 172108. https://doi.org/10.1063/1.2117630.
